# Acute and midterm outcomes of patients undergoing right-sided heart valve surgery for carcinoid heart valve disease

**DOI:** 10.1016/j.xjtc.2026.102326

**Published:** 2026-03-24

**Authors:** Harun Sarwari, Simon Pecha, Yalin Yildirim, Christoph Sinning, Niklas Schofer, Andreas Schaefer, Evaldas Girdauskas, Hermann Reichenspurner, Yousuf Al Assar, Johannes Petersen

**Affiliations:** a Department of Cardiovascular Surgery, University Heart & Vascular Center Hamburg, Hamburg, Germany; b DZHK (German Centre for Cardiovascular Research), Partner Site North, Hamburg, Germany; c Department of Cardiology, University Heart & Vascular Center Hamburg, Hamburg, Germany

**Keywords:** neuroendocrine tumor, carcinoid heart syndrome, right heart failure, tricuspid valve replacement, pulmonary valve replacement, transcatheter valve-in-valve intervention

## Abstract

**Objectives:**

Carcinoid heart syndrome is a rare manifestation of metastatic neuroendocrine tumor that causes right heart valve dysfunction and subsequent symptomatic right heart failure, reducing long-term survival. In addition to symptomatic treatment, therapeutic strategies include surgical or interventional valve replacement for tricuspid and pulmonary valve regurgitation.

**Methods:**

Between 2011 and 2022, 12 patients with symptomatic right heart failure and carcinoid heart syndrome underwent elective valve surgery at our institution and were retrospectively included in this study. Procedural data, early clinical outcomes, survival, and time to reintervention were analyzed. The median follow-up period was 33 months, ranging from 2 to 131 months.

**Results:**

All patients underwent tricuspid bioprosthetic valve replacement with concomitant pulmonary valve replacement in 4 patients. A 3-dimensional endoscopic approach via right anterolateral minithoracotomy was conducted in 33.3% (4 out of 12) of cases, whereas a full median sternotomy was performed in 66.7% (8 out of 12). Beating-heart technique without crossclamping of the aorta was used in 58.3% (7 out of 12) of cases. Postoperative rethoracotomy for bleeding was required in 2 patients, and 1 patient developed acute kidney injury. No patients experienced permanent pacemaker implantation or stroke. One patient died during hospitalization due to acute hepatic failure in the presence of a preoperatively elevated Model for End-Stage Liver Disease score of 13, resulting in an in-hospital mortality rate of 8.3%. At final follow-up, overall survival was 41.7% (5 out of 12). Five patients required valve reintervention due to bioprosthetic degeneration associated with progression of the underlying neuroendocrine tumor, occurring 11 to 44 months after the index procedure. Two patients underwent surgical redo procedures (1 repeat tricuspid valve replacement and 1 repeat pulmonary valve replacement). One patient underwent pulmonary balloon valvuloplasty followed by 2 valve-in-valve procedures using balloon-expandable transcatheter heart valves. In addition, 2 patients underwent combined valve-in-valve interventions of both the tricuspid and pulmonary valves. Four of the 5 patients undergoing reintervention died during subsequent follow-up.

**Conclusions:**

The results from our single-center study provide descriptive information on current treatment approaches for this rare cardiovascular disease. Surgical valve replacement for carcinoid heart syndrome is technically feasible and associated with satisfactory early clinical outcomes. However, reinterventions are frequently required, largely driven by progression of neuroendocrine tumor disease. Consequently, the influence of valve intervention on long-term survival remains uncertain, and valve durability appears limited, as reflected by the high rate of repeat procedures. These findings underscore the necessity for close follow-up with dedicated heart valve units, enabling early detection of disease progression and bioprosthetic valve degeneration and ensuring optimized care through a multidisciplinary treatment approach.

**Figure:**
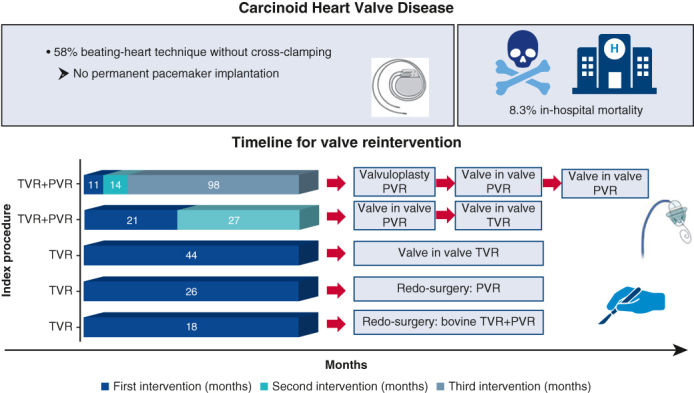
Kaplan-Meier survival and timeline from index procedure to cardiac reintervention in NET.

**Central Message**

Management of carcinoid heart valve disease requires a multidisciplinary heart team. Surgery ensures early success, but tumor progression often demands transcatheter reinterventions.

**Perspective**

Management of carcinoid heart valve disease requires a multidisciplinary approach, with surgeons and cardiologists collaborating closely. Surgical valve replacement offers good early results, but tumor progression may require reinterventions such as valve-in-valve therapy. Optimal long-term management is delivered by teams combining surgical, transcatheter, and oncological expertise.

Neuroendocrine tumors (NETs) represent a diverse group of neoplasms that can metastasize throughout the body.^1^ Although NETs are generally considered rare, their incidence has been gradually increasing. The clinical significance is largely determined by their potential for metastatic spread and secretion of bioactive substances, which can result in distinctive clinical syndromes (eg, Zollinger-Ellison syndrome, insulinoma syndrome, and somatostatinoma syndrome).^2^^,^^3^ One particularly challenging manifestation is carcinoid heart syndrome (CHS). In this condition, vasoactive substances such as serotonin are released into the bloodstream, triggering a cascade of events that significantly influence the cardiovascular system. These effects include fibrotic remodeling of the heart valves, primarily affecting the tricuspid valve (TV) and pulmonary valve (PV), leading to valvular dysfunction with severe regurgitation and symptomatic right heart failure.^4^^,^^5^ Managing CHS requires a multidisciplinary approach involving oncologists, cardiologists, and cardiac surgeons to address the complex interplay of its pathogenesis and progression. Valve surgery plays a crucial role in this treatment strategy, aiming to alleviate symptoms of right heart failure, preserve cardiac function, and improve overall quality of life.^6^, ^7^, ^8^

Nevertheless, given the progressive nature of NETs, the long-term prognostic influence of valve intervention remains uncertain. Herein, we report acute and midterm outcomes of patients undergoing right-sided valve surgery for CHS in a descriptive, hypothesis-generating analysis.

## Methods

### Ethical Statement

In view of the fact that the patient data that are the subject of the study can no longer be attributed to a human being, the study project does not constitute a “research project involving human beings” as defined in Section 9^2^ of the Hamburg Chamber Act for the Medical Professions and also does not fall within the scope of the research projects requiring consultation pursuant to Section 15^1^ of the Professional Code of Conduct for Hamburg Physicians. Thus, the study project does not require consultation with the Ethics Committee of the Hamburg Medical Association. Informed patient consent was waived by the ethics board.

### Perioperative Strategy

The hospital employed a multidisciplinary team approach for each case, consisting of oncologists, cardiac surgeons, endocrinologists, cardiologists, and anesthesiologists. Following the acceptance of each patient for cardiac surgery, a standardized medical and surgical protocol was defined. All cases received an octreotide infusion (100 μg/hour, 12 hours before surgery) which was continued for the first 48 hours postsurgery. Subsequent to this, patients were administered their long-acting somatostatin analogue. In addition to the octreotide infusion, bolus infusions of steroids and antihistamines were also administered to treat the pharmacological symptoms of a carcinoid crisis, which included tachycardia, bronchospasm, flushing, and labile blood pressure.

### Intraoperative Strategy

**Figure 1:**
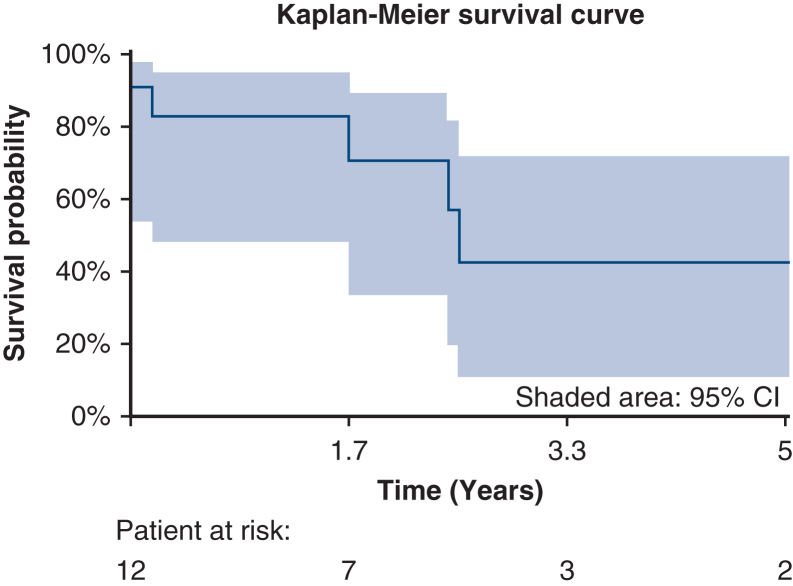
Kaplan-Meier curves depicting long-term survival in all patients. Kaplan-Meier analysis showing a 41.7% survival rate at 5 years.

Replacement of the TV and PV were indicated due to the characteristic fibrotic changes associated with carcinoid heart disease resulting in leaflet thickening, retraction, and restricted mobility (Figure 1). Depending on the extent of valvular involvement, combined TV replacement (TVR) and PV replacement (PVR) was performed via median sternotomy, using arterial cannulation of the ascending aorta and bicaval venous cannulation. For isolated TVR, a right anterolateral minithoracotomy was routinely employed, utilizing a soft tissue retractor and a fully endoscopic 3-dimensional approach. In the absence of an atrial septal defect or a persistent foramen ovale, a beating-heart technique was applied without aortic crossclamping.

### Patient Selection and Data Collection

This single-center retrospective study evaluated the procedural and clinical outcomes, survival rates, and reintervention times of 12 patients diagnosed with CHS who underwent elective cardiac surgery at our institution between June 2011 and April 2022. All patients were diagnosed with NETs either via biopsy or histological examination. CHS was diagnosed based on clinical presentation and echocardiographic findings. Surgery was performed after evaluation by a multidisciplinary structural heart team. Preoperative hepatic function was assessed retrospectively using the Model for End-Stage Liver Disease (MELD) score to better characterize operative risk in this patient population. Data collection and patient management adhered to institutional ethical standards in accordance with the Declaration of Helsinki.

Comprehensive data were retrieved from electronic health records, including demographic characteristics, primary tumor location, metastatic pattern, and clinical symptomatology. Detailed echocardiographic evaluations were performed to assess the extent and severity of valvular involvement. Surgical treatment strategies were documented, along with perioperative outcomes and follow-up data. Follow-up duration was tracked meticulously for all patients, allowing assessment of long-term outcomes, including survival, and time to reintervention.

### Statistical Analysis

Descriptive statistics were used with continuous variables presented as means with SD and categorical variables as frequencies and percentages. To evaluate short-term and long-term survival rates following surgery, a Kaplan-Meier curve was used to analyze time-to-event data.

## Results

### Patient Characteristics

Baseline characteristics are outlined in Table 1. Mean age was 62.0 ± 12.4 years, with 75% of the patients being men. Mean body mass index was 25.7 ± 3.8. European System for Cardiac Operative Risk Evaluation II score was 1.1% ± 0.4%, and Society of Thoracic Surgeons score was 2.5% ± 1.6%. None of the patients had undergone a previous cardiac surgery.

**Table 1:** Preoperative baseline characteristics

Baseline characteristic	Study group (n = 12)
Age (y)	62.0 ± 12.4
Male sex	9 (75.0)
BMI	25.7 ± 3.8
Previous sternotomy	0 (0)
EuroSCORE II (%)	1.1 ± 0.4
STS score (%)	2.5 ± 1.6
Coronary artery disease	3 (25.0)
proBNP (ng/L)	884.3 ± 513.0
Hyperlipidemia	1 (8.3)
Atrial fibrillation	0 (0)
Pacemaker	0 (0)
Arterial hypertension	3 (25.0)
Diabetes	0 (0)
Stroke	0 (0)
Creatinine (mg/dL)	1.1 ± 0.3
Extracardiac artheropathy	0 (0)
Pulmonary hypertension	0 (0)
COPD	0 (0)
MELD score	8.8 ± 3.7

Values are presented as mean ± SD, or n (%). *BMI*, Body mass index; *EuroSCORE*, European System for Cardiac Operative Risk Evaluation; *STS*, Society of Thoracic Surgeons; *proBNP*, pro brain natriuretic peptide; *COPD*, chronic obstructive pulmonary disease; *MELD*, Model for End-Stage Liver Disease.

Median interval between the initial diagnosis of NETs and the development of CHS was 11 months (range, 1-72 months). All patients had hepatic metastases at the time of surgery, with a mean MELD score of 8.8 ± 3.7, reflecting moderate hepatic dysfunction. Additional metastatic involvement included lymphatic (58.3%), pulmonary and osseous (16.7%), and ovarian (8.3%) sites.

**Figure 2:**
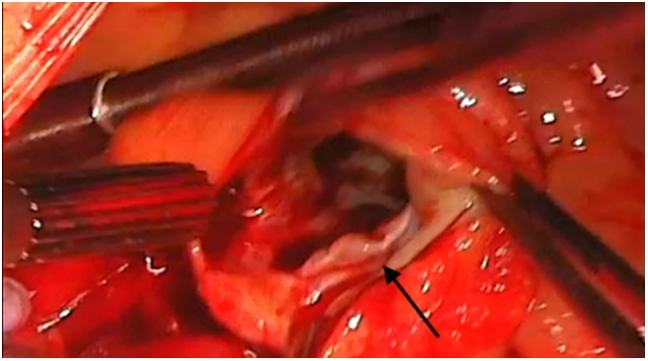
This image shows the pulmonary valve during surgery in a patient with carcinoid heart syndrome (*CHS*). The valve demonstrates the typical fibrotic thickening and retraction characteristic of CHS, resulting in stiff and dysfunctional leaflets.

Preoperative echocardiography demonstrated severe TV regurgitation with characteristic fibrotic retraction of the valve leaflets in all patients (Figure 2) . In addition, 33.3% (4 out of 12) of patients had severe PV regurgitation, and 25% (3 out of 12) presented with PV stenosis requiring additional surgical intervention. Mean left ventricular ejection fraction was 57.3% ± 5.2%, mean tricuspid annular plane systolic excursion was 23.2 ± 4.8 mm, and mean systolic pulmonary artery pressure was 27.7 ± 8.6 mm Hg (Table 2).

**Table 2:** Echocardiographic findings

Echocardiography parameter	Study group (n = 12)
TAPSE (mm)	23.2 ± 4.8
sPAP (mm Hg)	27.7 ± 8.6
LVEF (%)	57.3 ± 5.2
Tricuspid valve regurgitation ≥III	12 (100)
Pulmonary valve regurgitation	
≥III	3 (25.0)
II	4 (33.3)

Values are presented as mean ± SD, or n (%). *TAPSE*, Tricuspid annular plane systolic excursion; *sPAP*, systolic pulmonary arterial pressure; *LVEF*, left ventricular ejection fraction.

### Surgical Management

All 12 patients underwent TVR, with 4 requiring concomitant PVR. Combined procedures were performed through a median sternotomy. Of 8 patients who underwent isolated TVR, 50% were provided with a 3-dimensional fully endoscopic approach via right anterolateral minithoracotomy. Seven patients underwent cardiac surgery using beating heart technique without crossclamping of the aorta. Among these, 3 received TVR and PVR, whereas 4 received isolated TVR. Cardiopulmonary bypass and aortic crossclamp times were 135.3 ± 98.4 minutes and 98.2 ± 41.8 minutes, respectively. For TVR, 7 stented bovine bioprostheses (58.3%) and 5 stented porcine bioprostheses (41.7%) were used. For PVR, stented bovine bioprostheses were used exclusively (detailed surgical management is shown in Table 3).

**Table 3:** Surgical management

Periprocedural data	Study group (n = 12)
Surgical access	
Median sternotomy	8 (66.7)
Minimally invasive access	4 (33.3)
Beating heart	7 (58.3)
Aortic crossclamp time (min)	98.2 ± 41.8
Cardiopulmonary bypass time (min)	135.3 ± 98.4
Cardiopulmonary bypass time: isolated (min)	113.5 ± 31.9
Cardiopulmonary bypass time: concomitant (min)	204.0 ± 131.6
Procedure duration (min)	220.9 ± 99.5

Values are presented as n (%) or mean ± SD.

### Clinical Outcomes and Follow-up

**Figure 3:**
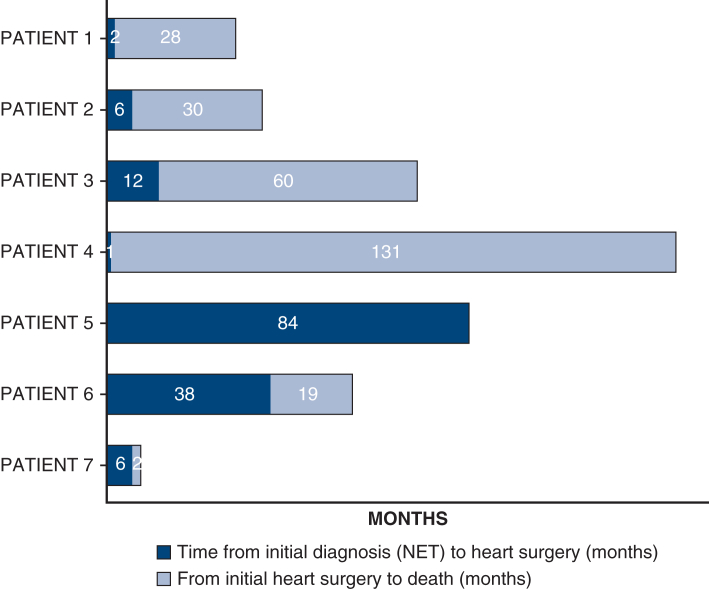
Timeline from initial diagnosis of neuroendocrine tumor (*NET*) until surgery and death. The bar chart illustrates the timeline from the initial diagnosis of NET until valve surgery and death for the 7 patients who died. The blue bars represent the time in months from the initial NET diagnosis to the valve surgery. The gray bars indicate the duration in months from the heart surgery until death.

Clinical outcomes are summarized in Table 4. One patient died during the index hospitalization from acute hepatic failure in the setting of a preoperatively elevated MELD score of 13, resulting in an in-hospital mortality rate of 8.3%. There were no cases of perioperative myocardial infarction or disabling stroke. Bleeding complications occurred in 16.6% (2 out of 12) of patients. One patient (8.3%) developed acute kidney injury (Acute Kidney Injury Network stage 2-3) postoperatively. No patient required permanent pacemaker implantation. The median follow-up period was 33 months (range, 2-131 months). During follow-up, 6 additional patients died due to complications related to the progression of their underlying NETs, resulting in an overall mortality rate of 58.3% (7 out of 12). Consequently, the overall survival rate at last follow-up was 41.7% (5 out of 12) (Figure 1). The interval between the initial valve surgery and death varied considerably, highlighting the heterogeneity of disease progression in this patient population (Figure 3). In 5 of the 7 patients who died, the time from NET diagnosis to cardiac surgery was relatively short, ranging from 1 month (patient 4) to 84 months (patient 5). The postoperative survival time also showed substantial variability, with patient 4 achieving the longest survival (131 months) and patient 7 the shortest (2 months). In the remaining 2 patients who died, cardiac surgery was performed 38 and 84 months after NET diagnosis, with subsequent survival times of 0 and 19 months, respectively.

**Table 4:** Clinical outcomes

Clinical outcome	Study group (n = 12)
In-hospital mortality	1 (8.3)
Myocardial infarction	0 (0)
Disabling stroke	0 (0)
Bleeding complication	2 (16.6)
Acute kidney injury: AKIN 2-3	1 (8.3)
Pacemaker implantation	0 (0)

Values are presented as n (%). *AKIN*, Acute Kidney Injury Network.

### Reintervention During Follow-up

Due to NET progression and subsequent bioprosthetic valve degeneration, 5 patients required valve reintervention between 11 and 44 months after the index procedure. Surgical findings during redo procedures demonstrated mixed patterns of valve degeneration, characterized by fibrotic leaflet restriction and stenotic changes, rather than isolated calcification or thrombosis.

**Figure 4:**
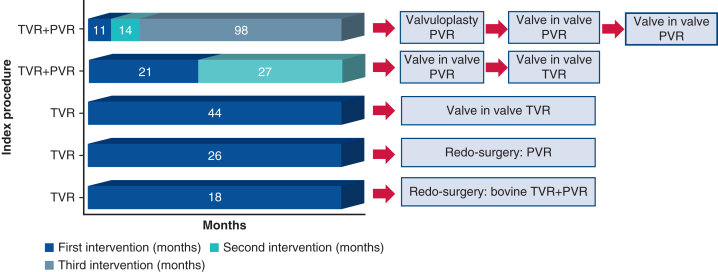
Timeline from index procedure to cardiac reintervention due to neuroendocrine tumor (*NET*) progression. The bar chart illustrates the timeline from the index procedure to cardiac reintervention due to neuroendocrine tumor (*NET*) progression for 5 affected patients. On the *y*-axis, the initial index procedures are listed, whereas the bars represent the time span in months until reinterventions were performed. Different colors within the bars distinguish multiple reinterventions for individual patients. Each patient underwent 1 or more cardiac reinterventions, which are displayed next to the respective bars. *TVR*, Tricuspid valve replacement; *PVR*, pulmonary valve replacement.

Among these patients, 2 underwent repeat surgical interventions (1 redo TVR and 1 redo PVR). One patient underwent pulmonary balloon valvuloplasty followed by 2 valve-in-valve (ViV) procedures using balloon-expandable transcatheter heart valves. In addition, 2 patients underwent combined ViV interventions of both the TV and PV using balloon-expandable transcatheter heart valves (Figure 4). During subsequent follow-up, 4 of the 5 patients who underwent reintervention died: 2 within 31 and 49 days, respectively, and 2 at 8 and 15 months after the final reintervention. One patient remained alive at final follow-up.

## Discussion

This study demonstrates that right-sided heart valve surgery for CHS is technically feasible and can achieve good early outcomes, even in this complex patient group. However, beyond technical feasibility, it is essential to place these findings in the context of current treatment strategies and disease-specific challenges. Management of CHS remains difficult due to the combination of severe valve damage and the progressive nature of NETs.^9^, ^10^, ^11^ According to current guidelines, treatment should be tailored to the patient's overall disease status, valvular pathology, and surgical risk.^12^ In patients with noncarcinoid valve disease, valve repair is generally preferred to preserve native structures. In CHS, extensive leaflet fibrosis usually makes repair impossible, and transcatheter edge-to-edge techniques are often ineffective due to restricted leaflet motion. Therefore, current guidelines recommend valve replacement, either surgical or transcatheter, as the standard of care for most patients with CHS.^12^^,^^13^

Historically, surgical outcomes in CHS were poor, with perioperative mortality exceeding 20% before the 1990s. Advances in surgical techniques, anesthesia, and multidisciplinary care have significantly improved results, with recent data reporting perioperative mortality rates below 5%.^14^ In our series, the in-hospital mortality was 8.3%, which is comparable to other reports of TV and PV surgery in patients without CHS.^15^ However, the observed death was likely driven by the presence of preexisting liver dysfunction, as indicated by a preoperative MELD score of 13—a level of hepatic impairment that has been shown to adversely influence outcomes after cardiac surgery. Preoperative hepatic dysfunction quantified by the MELD score has been independently associated with increased perioperative mortality and major postoperative complications in patients undergoing cardiac procedures, with higher scores conferring significantly higher operative mortality compared with patients with lower MELD values.^16^ Nonetheless, when viewed in the context of a recent study analyzing patients with carcinoid syndrome (n = 11), the 30-day mortality rate in that cohort exceeded 17%, underscoring the particularly high risk in this specific population.^17^ We believe that the lower mortality rate in our study was caused by the long-standing presence of an interdisciplinary heart team in our institution. Furthermore, our results demonstrated that overall perioperative complications were low, and no patient required permanent pacemaker implantation—an important finding given that TVRs are often associated with conduction disturbances, with pacemaker implantation rates reported as high as 27%.^18^^,^^19^

We believe this low rate of conduction-related complications is related to the routine use of the beating-heart technique. A key advantage of the beating-heart approach is the avoidance of cardioplegia while allowing real-time monitoring of conduction. In our cohort, if a suture was placed too close to the conduction system near Koch's triangle and caused a conduction disturbance on the electrocardiogram, it was immediately recognized and corrected by adjusting or removing the suture. This intraoperative correction likely contributed to the absence of permanent pacemaker implantation.

Despite favorable perioperative results, our study confirms that long-term durability remains a major concern. Bioprosthetic valves are prone to early degeneration due to ongoing NET activity. This is consistent with current evidence showing that patients with persistent NET disease have a higher risk of accelerated bioprosthetic failure.^20^^,^^21^ In our cohort, 5 patients required repeat intervention within 4 years of the initial procedure, and survival after reintervention was poor—4 of these 5 patients died—highlighting that despite the availability of reinterventions, long-term survival remains uncertain and valve durability is limited with frequent need for repeat procedures.

Although mechanical valves offer superior durability to bioprostheses, there is no high-level evidence suggesting improved outcomes compared with bioprosthetic valves in patients with CHS. We avoid mechanical valves in these patients to avoid obligatory lifelong anticoagulation therapy and its associated increased risk of bleeding in patients with liver metastases or those receiving systemic NET treatments.^20^

Consequently, valve selection in this cohort predominantly favored bioprosthetic valves based on surgical preference. In line with this strategy, current guidelines increasingly endorse transcatheter ViV procedures as a viable treatment option for degenerated right-sided bioprostheses, offering favorable short-term outcomes and reduced procedure-related risk compared with repeat open surgery.^21^, ^22^, ^23^, ^24^, ^25^, ^26^ In our cohort, 3 patients were successfully treated with ViV interventions in the tricuspid or pulmonary position, underscoring both the feasibility of this approach and the importance of management by a dedicated multidisciplinary heart team.

At the end of the follow-up, overall survival was 41.7%, which reflects mortality mainly due to progression of the underlying NET rather than cardiac causes. Overall mortality was driven by progressive NET disease and global cardiac decompensation rather than isolated valve-related causes, underscoring the systemic nature of CHS. This highlights the systemic nature of the disease and the need for close cooperation between oncologists and cardiologists. Optimal timing of surgery, consistent oncologic control, and structured follow-up are crucial to detect valve degeneration early and to plan reinterventions in a timely manner.

Right-sided valve replacement for CHS can be performed with acceptable perioperative risk when contemporary surgical strategies, such as the beating-heart technique, are applied. Preoperative assessment of hepatic function appears to be a critical determinant of clinical outcome, particularly with regard to perioperative mortality. Given the high incidence of bioprosthetic valve degeneration, transcatheter ViV interventions represent an important adjunct within the therapeutic pathway. Long-term management should be provided in centers with expertise in valvular heart disease and NETs to optimize outcomes and enable timely treatment of tumor progression.

### Limitations

Because this study reflects the experience of a single center, the generalizability of its findings to other institutions or settings is limited, particularly given the small sample size and no control group, which necessitates cautious interpretation of conclusions. Small-number bias limits interpretation, particularly of survival curves and subgroup analyses. A multicenter analysis could provide further insights and enhance the robustness of these findings. Additionally, the follow-up period in this study is limited to a median of 33 months (range, 2 to 131 months), which also necessitates careful consideration when drawing conclusions.

## Conclusions

The results from our single-center study offer valuable insights into the available treatment options for this rare cardiovascular disease. Conversely, right-sided heart valve surgery in CHS has been demonstrated to yield favorable acute outcomes without the necessity for pacemaker implantation, particularly when performed via the beating-heart technique. Impaired preoperative hepatic function has been associated with adverse clinical outcomes, with a particular influence on perioperative mortality. In view of the variability in disease progression and the risk of bioprosthetic valve degeneration, lifelong surveillance with a dedicated heart valve unit is imperative. A multidisciplinary team approach is essential to ensure timely detection of tumor progression and valve dysfunction, and to provide optimal individualized care for this complex patient population.

## Conflict of Interest Statement

The authors reported no conflicts of interest.

The *Journal* policy requires editors and reviewers to disclose conflicts of interest and to decline handling or reviewing manuscripts for which they may have a conflict of interest. The editors and reviewers of this article have no conflicts of interest.

## References

[1] Halperin D.M., Shen C., Dasari A. (2017). Frequency of carcinoid syndrome at neuroendocrine tumour diagnosis: a population-based study. Lancet Oncol.

[2] Dasari A., Shen C., Halperin D. (2017). Trends in the incidence, prevalence, and survival outcomes in patients with neuroendocrine tumors in the United States. JAMA Oncol.

[3] Klöppel G. (2011). Classification and pathology of gastroenteropancreatic neuroendocrine neoplasms. Endocr Relat Cancer.

[4] Hayes A.R., Davar J., Caplin M.E. (2018). Carcinoid heart disease: a review. Endocrinol Metab Clin North Am.

[5] Pellikka P.A., Tajik A.J., Khandheria B.K. (1993). Carcinoid heart disease: clinical and echocardiographic spectrum in 74 patients. Circulation.

[6] Bhattacharyya S., Raja S.G., Toumpanakis C., Caplin M.E., Dreyfus G.D., Davar J. (2011). Outcomes, risks, and complications of cardiac surgery for carcinoid heart disease. Eur J Cardiothorac Surg.

[7] Mokhles P., van Herwerden L.A., de Jong P.L. (2012). Carcinoid heart disease: outcomes after surgical valve replacement. Eur J Cardiothorac Surg.

[8] Edwards N.C., Yuan M., Nolan O. (2016). Effect of valvular surgery in carcinoid heart disease: an observational cohort study. J Clin Endocrinol Metab.

[9] Møller J.E., Connolly H.M., Rubin J., Seward J.B., Modesto K., Pellikka P.A. (2003). Factors associated with the progression of carcinoid heart disease. N Engl J Med.

[10] Lundin L., Norheim I., Landelius J., Oberg K., Theodorsson-Norheim E. (1988). Carcinoid heart disease: relationship of circulating vasoactive substances to ultrasound-detectable cardiac abnormalities. Circulation.

[11] Robiolio P.A., Rigolin V.H., Wilson J.S., Harrison J.K., Sanders L.L., Bashore T.M. (1995). Carcinoid heart disease: correlation of high serotonin levels with valvular abnormalities detected by cardiac catheterization and echocardiography. Circulation.

[12] Vahanian A., Beyersdorf F., Praz F. (2022). 2021 ESC/EACTS guidelines for the management of valvular heart disease. Eur Heart J.

[13] Möllmann H., von Bardeleben R.S., Dreger H. (2022). Trikuspidalklappeninsuffizienz: DGK-Positionspapier. Kardiologie.

[14] Nguyen A., Schaff H.V., Abel M.D. (2019). Improving outcome of valve replacement for carcinoid heart disease. J Thorac Cardiovasc Surg.

[15] Mahboobi S.K., Sharma S., Ahmed A.A. (2025). StatPearls [Internet].

[16] Pathare P., Elbayomi M., Weyand M., Griesbach C., Harig F. (2023). MELD score for risk stratification in cardiac surgery. Heart Vessels.

[17] El Gabry M., Arends S., Shehada S.E. (2023). Hedinger syndrome—lessons learnt: a single-center experience. J Cardiovasc Dev Dis.

[18] Fu W., Wagner C.M., Brescia A.A. (2024). Pacemaker implantation after tricuspid valve surgery at a high-volume regional reference center. Ann Thorac Surg Short Rep.

[19] Kassab J., Harb S.C., Desai M.Y. (2024). Incidence, risk factors, and outcomes associated with permanent pacemaker implantation following tricuspid valve surgery. J Am Heart Assoc.

[20] Møller J.E., Pellikka P.A., Bernheim A.M., Schaff H.V., Rubin J., Connolly H.M. (2005). Prognosis of carcinoid heart disease: analysis of 200 cases over two decades. Circulation.

[21] Korach A., Grozinsky-Glasberg S., Atlan J. (2016). Valve replacement in patients with carcinoid heart disease: choosing the right valve at the right time. J Heart Valve Dis.

[22] Dannenberg V., Donà C., Koschutnik M. (2021). Transcatheter treatment by valve-in-valve and valve-in-ring implantation for prosthetic tricuspid valve dysfunction. Wien Klin Wochenschr.

[23] Chandavimol M., Ngernsritrakul T., Meemook K. (2021). Transcatheter tricuspid valve-in-valve implantation for degenerative surgical bioprosthesis using SAPIEN 3: a case series. Clin Case Rep.

[24] McElhinney D.B., Cabalka A.K., Aboulhosn J.A. (2016). Transcatheter tricuspid valve-in-valve implantation for the treatment of dysfunctional surgical bioprosthetic valves: an international, multicenter registry study. Circulation.

[25] Wu K., Shen J., Meng X. (2024). Transcatheter valve-in-valve implantation treatment with the J-valve system for tricuspid bioprosthesis deterioration: a report of two cases. J Thorac Dis.

[26] Schaefer A., Demal T.J., Bhadra O.D. (2023). Valve-in-valve procedures for degenerated surgical and transcatheter aortic valve bioprostheses using a latest-generation self-expanding intra-annular transcatheter heart valve. Front Cardiovasc Med.

